# Relationship between attitude towards vaccination against COVID-19 and psychological characteristics of personality

**DOI:** 10.1192/j.eurpsy.2022.500

**Published:** 2022-09-01

**Authors:** O. Boyko, T. Medvedeva, S. Enikolopov, O. Vorontsova, O. Kazmina

**Affiliations:** Federal State Budgetary Scientific institution “Mental health research center”, Clinical Psychology, Moscow, Russian Federation

**Keywords:** vaccination, attitude, Covid-19

## Abstract

**Introduction:**

Vaccination is an effective way to control the infection. COVID-19 is a new disease, and so is the vaccine against it.

**Objectives:**

The aim of the study was to investigate psychological characteristics associated with attitude towards vaccination.

**Methods:**

An online survey was used (N=1336) (31.03.2020–9.02.2021). Respondents completed COPE, «Moral dilemmas» (30 Green’s Dilemmas, 10 of each type) and decided which strategy to stop the pandemic they found the effective (vaccination, herb immunity, innovative treatment or simply waiting until it fades away on its own). The study analyzed groups of those who see the benefits of vaccination and those who do not consider vaccination as a way to solve the problem of coronavirus.

**Results:**

«Vaccination» attitude is more typical for men, for younger people and is also associated with assessment of COVID-19 as a dangerous disease (61% versus 21% for «vaccination» and «no vaccination» groups respectively), more diligent compliance with anti-epidemic rules (3,7 and 2,9 mean number of protection methods used), at the same time, the “vaccination” group responds about the less inconvenience associated with restrictions during the pandemic. This social attitude is associated with «need for creativity» and constructive coping: «planning», «concentration on emotions», the use of instrumental and emotional social support. There is a difference in personal moral choices (3,6 versus 2,9 for «vaccination» and «no vaccination» groups respectively), that demonstrated that positive attitude towards vaccination signifies an active personal position.

**Conclusions:**

Positive attitude towards vaccination is associated with a proactive personal position and involvement in social interaction using interpersonal coping strategies.

**Disclosure:**

No significant relationships.

